# (*E*)-1-(2,5-Dichloro­thio­phen-3-yl)ethan­one [8-(trifluoro­meth­yl)quinolin-4-yl]hydrazone

**DOI:** 10.1107/S1600536812005673

**Published:** 2012-02-24

**Authors:** A. S. Dayananda, H. S. Yathirajan, William T. A. Harrison, Alexandra M. Z. Slawin

**Affiliations:** a Department of Studies in Chemistry, University of Mysore, Manasagangotri, Mysore 570 006, India; b Department of Chemistry, University of Aberdeen, Aberdeen AB24 3UE, Scotland; c School of Chemistry, University of St Andrews, St Andrews KY16 9ST, Scotland

## Abstract

In the title compound, C_16_H_10_Cl_2_F_3_N_3_S, the dihedral angle between the quinoline and thio­phene ring systems is 4.94 (10)°. The NH group of the hydrazone moiety does not form a hydrogen bond, due to a steric crowding. In the crystal, the thio­phene ring takes part in weak π–π stacking inter­actions with the pyridine ring [centroid-to-centroid separation = 3.7553 (19) Å and inter­planar angle = 5.48 (12)°] and the benzene ring [3.7927 (19) Å and 4.58 (12)°]. Together, these lead to [100] stacks of mol­ecules in an alternating head-to-tail arrangement, with two π–π stacking contacts between each adjacent pair.

## Related literature

For related structures derived from 4-hydrazinyl-8-(trifluoro­meth­yl)quinoline and background to Schiff bases, see; Jasinski *et al.* (2010 ▶); Dutkiewicz *et al.* (2010 ▶).
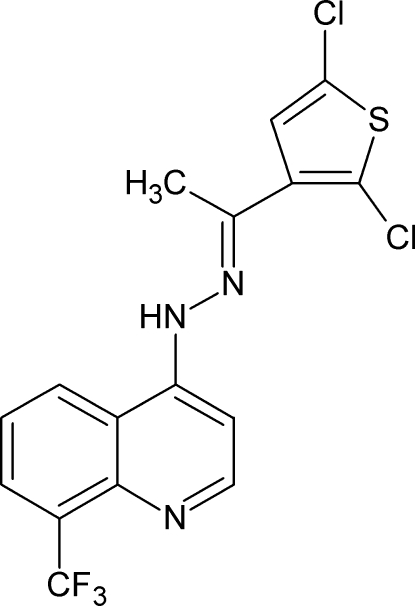


## Experimental

### 

#### Crystal data

C_16_H_10_Cl_2_F_3_N_3_S
*M* *_r_* = 404.23
Monoclinic, 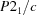

*a* = 7.687 (2) Å
*b* = 14.392 (5) Å
*c* = 14.360 (5) Å
β = 95.053 (9)°
*V* = 1582.5 (9) Å^3^
*Z* = 4
Mo *K*α radiation
μ = 0.58 mm^−1^
*T* = 73 K
0.10 × 0.10 × 0.10 mm

#### Data collection

Rigaku Mercury CCD diffractometer
9805 measured reflections
2897 independent reflections
2369 reflections with *I* > 2σ(*I*)
*R*_int_ = 0.082

#### Refinement

*R*[*F* ^2^ > 2σ(*F* ^2^)] = 0.049
*wR*(*F* ^2^) = 0.128
*S* = 1.06
2897 reflections
231 parameters
H atoms treated by a mixture of independent and constrained refinement
Δρ_max_ = 0.35 e Å^−3^
Δρ_min_ = −0.37 e Å^−3^

### 

Data collection: *CrystalClear* (Rigaku, 2009 ▶); cell refinement: *CrystalClear*; data reduction: *CrystalClear*; program(s) used to solve structure: *SHELXS97* (Sheldrick, 2008 ▶); program(s) used to refine structure: *SHELXL97* (Sheldrick, 2008 ▶); molecular graphics: *ORTEP-3* (Farrugia, 1997 ▶); software used to prepare material for publication: *SHELXL97*.

## Supplementary Material

Crystal structure: contains datablock(s) global, I. DOI: 10.1107/S1600536812005673/kp2384sup1.cif

Structure factors: contains datablock(s) I. DOI: 10.1107/S1600536812005673/kp2384Isup2.hkl

Supplementary material file. DOI: 10.1107/S1600536812005673/kp2384Isup3.cml

Additional supplementary materials:  crystallographic information; 3D view; checkCIF report

## Figures and Tables

**Fig. 1.**
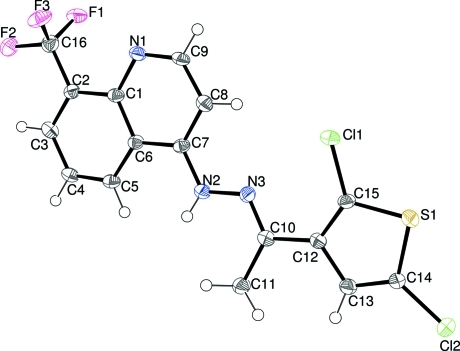
The molecular structure of (I) showing 30% probability displacement ellipsoids for non-H atoms.

**Fig. 2.**
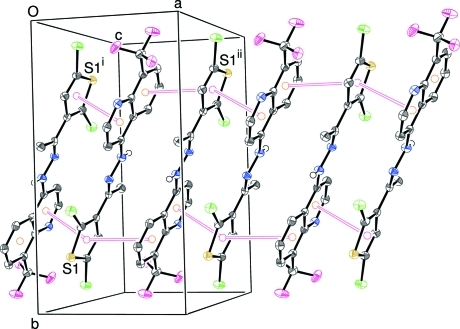
Part of a [100] chain of molecules linked by aromatic π–π stacking interactions. Symmetry codes: (i) -x, 1 - y, 1 - z; (ii) 1 - x, 1 - y, 1 - z.

## supplementary crystallographic information

### Comment

As a part of our ongoing studies of Schiff bases derived from 4-hydrazinyl-8-(trifluoromethyl)quinoline (Jasinski *et al.*, 2010; Dutkiewicz *et al.*, 2010), we now describe the synthesis and structure of the title compound, (I), (Fig. 1).

The quinoline ring system (C1–C9,N1) in (I) is almost planar (r.m.s. deviation = 0.026 Å). It subtends a dihedral angle of 4.94 (10)° with respect to the thiophene ring (C12–C15,S1). One F atom of the trifluoromethane group lies close to the quinoline plane [deviation = -0.098 (2) Å], whereas the other two F atoms are displaced by 1.034 (1) and -1.109 (2) Å. The two Cl atoms bonded to the thiophene ring are slightly displaced from the ring plane by almost the same magnitude but in opposite direction [-0.050 (1) Å for Cl1 and 0.045 (1) Å for Cl2].

In the crystal, the NH group does not form a hydrogen bond, as it seems to be sterically blocked by H5 and the C11 methyl group. π-π Stacking between the thiophene ring and the pyridine ring [(with symmetry operation -x, 1 - y, 1 - z), and centroid–centroid separation = 3.7553 (19) Å; interplanar angle = 5.48 (12)°] and also to a benzene ring [symmetry operated (1 - x, 1 - y, 1 - z) and centroid–centroid separation of 3.7927 (19) Å and interplanar angle of 4.58 (12)°] leads to [100] stacking of the molecules in an alternating head to tail arrangement. Each adjacent pair of molecules is linked by two π–π stacking interactions (Fig. 2).

### Experimental

A solution of 4-hydrazino-8-(trifluoromethyl)quinoline (2.2 g, 10 mmol) and 2,5-dichloro-3-acetylthiophene (1.99 g, 10.2 mmol) in 10 ml of ethanol was refluxed for 24 h under a nitrogen atmosphere in a dark. Then, the reaction mass was cooled and the solid separated by filtration. Colourless prisms of (I) were obtained by slow evaporation of an ethyl acetate solution (m.p. 465–467 K). Anal. Calcd. for C_16_H_10_Cl_2_F_3_N_3_S: C 47.54; H 2.49; N 10.39; S 7.93%; Found: C 47.51; H 2.48; N 10.36; S 7.91%.

### Refinement

The N-bound H atom was located in a difference map. Its position was freely refined with the constraint *U*_iso_(H) = 1.2*U*_eq_(N) applied. The C-bound H atoms were geometrically placed (C—H = 0.95-0.98Å) and refined as riding with *U*_iso_(H) = 1.2*U*_eq_(C).

### Crystal data

**Table:**

C_16_H_10_Cl_2_F_3_N_3_S	*F*(000) = 816
*M**_r_* = 404.23	*D*_x_ = 1.697 Mg m^−^^3^
Monoclinic, *P*2_1_/*c*	Mo *K*α radiation, λ = 0.71073 Å
Hall symbol: -P 2ybc	Cell parameters from 4944 reflections
*a* = 7.687 (2) Å	θ = 2.0–28.5°
*b* = 14.392 (5) Å	µ = 0.58 mm^−^^1^
*c* = 14.360 (5) Å	*T* = 73 K
β = 95.053 (9)°	Prism, colourless
*V* = 1582.5 (9) Å^3^	0.10 × 0.10 × 0.10 mm
*Z* = 4	

### Data collection

**Table:**

Rigaku Mercury CCD diffractometer	2369 reflections with *I* > 2σ(*I*)
Radiation source: fine-focus sealed tube	*R*_int_ = 0.082
Graphite monochromator	θ_max_ = 25.4°, θ_min_ = 2.0°
ω scans	*h* = −8→9
9805 measured reflections	*k* = −17→13
2897 independent reflections	*l* = −17→16

### Refinement

**Table:**

Refinement on *F*^2^	Secondary atom site location: difference Fourier map
Least-squares matrix: full	Hydrogen site location: inferred from neighbouring sites
*R*[*F*^2^ > 2σ(*F*^2^)] = 0.049	H atoms treated by a mixture of independent and constrained refinement
*wR*(*F*^2^) = 0.128	*w* = 1/[σ^2^(*F*_o_^2^) + (0.0544*P*)^2^ + 0.0123*P*] where *P* = (*F*_o_^2^ + 2*F*_c_^2^)/3
*S* = 1.06	(Δ/σ)_max_ < 0.001
2897 reflections	Δρ_max_ = 0.35 e Å^−^^3^
231 parameters	Δρ_min_ = −0.37 e Å^−^^3^
0 restraints	Extinction correction: *SHELXL97* (Sheldrick, 2008)
Primary atom site location: structure-invariant direct methods	

### Special details

**Table:**

Geometry. All esds (except the esd in the dihedral angle between two l.s. planes) are estimated using the full covariance matrix. The cell esds are taken into account individually in the estimation of esds in distances, angles and torsion angles; correlations between esds in cell parameters are only used when they are defined by crystal symmetry. An approximate (isotropic) treatment of cell esds is used for estimating esds involving l.s. planes.
Refinement. Refinement of F^2^ against ALL reflections. The weighted R-factor wR and goodness of fit S are based on F^2^, conventional R-factors R are based on F, with F set to zero for negative F^2^. The threshold expression of F^2^ > 2sigma(F^2^) is used only for calculating R-factors(gt) etc. and is not relevant to the choice of reflections for refinement. R-factors based on F^2^ are statistically about twice as large as those based on F, and R- factors based on ALL data will be even larger.

### Fractional atomic coordinates and isotropic or equivalent isotropic displacement parameters (Å^2^)

**Table:**

	*x*	*y*	*z*	*U*_iso_*/*U*_eq_	
C1	0.4410 (3)	0.25700 (19)	0.39915 (18)	0.0193 (6)	
C2	0.5208 (3)	0.16942 (18)	0.42084 (19)	0.0196 (6)	
C3	0.5718 (3)	0.1444 (2)	0.51114 (19)	0.0233 (6)	
H3	0.6244	0.0855	0.5241	0.028*	
C4	0.5458 (3)	0.20637 (19)	0.58497 (19)	0.0222 (6)	
H4	0.5840	0.1896	0.6474	0.027*	
C5	0.4662 (3)	0.29017 (19)	0.56742 (18)	0.0212 (6)	
H5	0.4475	0.3304	0.6180	0.025*	
C6	0.4113 (3)	0.31781 (19)	0.47515 (19)	0.0184 (6)	
C7	0.3276 (3)	0.40483 (18)	0.45162 (18)	0.0185 (6)	
C8	0.2886 (3)	0.4263 (2)	0.35855 (19)	0.0227 (6)	
H8	0.2367	0.4842	0.3407	0.027*	
C9	0.3269 (3)	0.3612 (2)	0.29090 (19)	0.0213 (6)	
H9	0.2985	0.3779	0.2274	0.026*	
C10	0.1600 (3)	0.59983 (19)	0.56281 (18)	0.0187 (6)	
C11	0.1918 (3)	0.5794 (2)	0.66590 (19)	0.0241 (6)	
H11A	0.1622	0.5145	0.6775	0.036*	
H11B	0.1188	0.6203	0.7007	0.036*	
H11C	0.3151	0.5902	0.6864	0.036*	
C12	0.0773 (3)	0.68922 (19)	0.53327 (18)	0.0181 (6)	
C13	0.0493 (3)	0.76274 (19)	0.59770 (19)	0.0210 (6)	
H13	0.0760	0.7573	0.6633	0.025*	
C14	−0.0183 (3)	0.83985 (18)	0.55647 (19)	0.0203 (6)	
C15	0.0230 (3)	0.71698 (18)	0.44394 (18)	0.0201 (6)	
C16	0.5511 (3)	0.1035 (2)	0.3424 (2)	0.0260 (7)	
N1	0.3989 (3)	0.27860 (16)	0.30701 (15)	0.0210 (5)	
N2	0.2884 (3)	0.46492 (16)	0.52193 (17)	0.0218 (5)	
H1	0.310 (3)	0.449 (2)	0.578 (2)	0.026*	
N3	0.2058 (3)	0.54653 (15)	0.49702 (15)	0.0205 (5)	
F1	0.65951 (18)	0.13668 (11)	0.28208 (10)	0.0273 (4)	
F2	0.6221 (2)	0.02239 (12)	0.37478 (12)	0.0380 (5)	
F3	0.40389 (19)	0.07936 (12)	0.28996 (12)	0.0349 (5)	
S1	−0.05583 (8)	0.82904 (5)	0.43743 (5)	0.0231 (2)	
Cl1	0.01844 (9)	0.65772 (5)	0.34012 (5)	0.0280 (2)	
Cl2	−0.06338 (9)	0.94308 (5)	0.60984 (5)	0.0289 (2)	

### Atomic displacement parameters (Å^2^)

**Table:**

	*U*^11^	*U*^22^	*U*^33^	*U*^12^	*U*^13^	*U*^23^
C1	0.0184 (12)	0.0234 (16)	0.0161 (14)	−0.0038 (11)	0.0015 (10)	−0.0023 (12)
C2	0.0190 (12)	0.0205 (16)	0.0196 (16)	−0.0017 (11)	0.0034 (11)	−0.0028 (12)
C3	0.0211 (13)	0.0273 (17)	0.0218 (16)	−0.0003 (11)	0.0026 (11)	0.0042 (13)
C4	0.0231 (13)	0.0282 (18)	0.0151 (15)	0.0004 (12)	0.0000 (11)	0.0022 (12)
C5	0.0220 (13)	0.0256 (17)	0.0156 (15)	−0.0015 (12)	0.0000 (11)	−0.0028 (12)
C6	0.0174 (13)	0.0203 (16)	0.0173 (15)	−0.0031 (11)	0.0007 (11)	−0.0022 (11)
C7	0.0165 (12)	0.0216 (16)	0.0173 (15)	−0.0027 (11)	0.0017 (10)	−0.0038 (11)
C8	0.0237 (13)	0.0241 (17)	0.0199 (16)	0.0004 (12)	0.0004 (11)	0.0024 (12)
C9	0.0233 (14)	0.0282 (17)	0.0118 (14)	−0.0020 (12)	−0.0017 (11)	0.0002 (12)
C10	0.0151 (12)	0.0232 (16)	0.0174 (15)	−0.0018 (11)	0.0000 (10)	0.0002 (12)
C11	0.0275 (14)	0.0244 (17)	0.0202 (16)	0.0045 (12)	0.0008 (11)	−0.0006 (12)
C12	0.0172 (12)	0.0201 (15)	0.0166 (15)	−0.0036 (11)	0.0002 (10)	0.0001 (11)
C13	0.0239 (13)	0.0220 (16)	0.0170 (15)	−0.0014 (11)	0.0013 (11)	−0.0005 (12)
C14	0.0249 (14)	0.0184 (16)	0.0178 (15)	−0.0001 (11)	0.0025 (11)	−0.0007 (11)
C15	0.0241 (13)	0.0205 (16)	0.0158 (15)	−0.0029 (11)	0.0019 (11)	−0.0034 (12)
C16	0.0266 (14)	0.0247 (18)	0.0271 (17)	−0.0027 (13)	0.0049 (12)	−0.0022 (13)
N1	0.0237 (11)	0.0243 (14)	0.0148 (12)	−0.0025 (10)	0.0010 (9)	0.0000 (10)
N2	0.0274 (11)	0.0222 (14)	0.0154 (12)	0.0021 (10)	−0.0008 (10)	0.0006 (11)
N3	0.0238 (11)	0.0186 (13)	0.0185 (13)	0.0020 (9)	−0.0007 (9)	−0.0003 (10)
F1	0.0267 (8)	0.0366 (11)	0.0194 (9)	−0.0026 (7)	0.0063 (7)	−0.0057 (7)
F2	0.0598 (11)	0.0226 (10)	0.0330 (11)	0.0107 (8)	0.0116 (9)	−0.0022 (8)
F3	0.0309 (9)	0.0410 (12)	0.0332 (10)	−0.0128 (7)	0.0056 (7)	−0.0185 (8)
S1	0.0268 (4)	0.0227 (5)	0.0193 (4)	0.0011 (3)	−0.0002 (3)	0.0026 (3)
Cl1	0.0410 (4)	0.0286 (5)	0.0140 (4)	0.0020 (3)	−0.0001 (3)	−0.0011 (3)
Cl2	0.0384 (4)	0.0216 (5)	0.0276 (5)	0.0048 (3)	0.0070 (3)	−0.0021 (3)

### Geometric parameters (Å, º)

**Table:**

C1—N1	1.370 (3)	C10—C12	1.481 (4)
C1—C2	1.424 (4)	C10—C11	1.508 (4)
C1—C6	1.433 (4)	C11—H11A	0.9800
C2—C3	1.369 (4)	C11—H11B	0.9800
C2—C16	1.507 (4)	C11—H11C	0.9800
C3—C4	1.413 (4)	C12—C15	1.373 (4)
C3—H3	0.9500	C12—C13	1.434 (4)
C4—C5	1.366 (4)	C13—C14	1.341 (4)
C4—H4	0.9500	C13—H13	0.9500
C5—C6	1.412 (4)	C14—S1	1.715 (3)
C5—H5	0.9500	C14—Cl2	1.721 (3)
C6—C7	1.435 (4)	C15—Cl1	1.715 (3)
C7—C8	1.379 (4)	C15—S1	1.722 (3)
C7—N2	1.383 (3)	C16—F1	1.342 (3)
C8—C9	1.400 (4)	C16—F3	1.349 (3)
C8—H8	0.9500	C16—F2	1.353 (3)
C9—N1	1.323 (3)	N2—N3	1.368 (3)
C9—H9	0.9500	N2—H1	0.85 (3)
C10—N3	1.290 (3)		
			
N1—C1—C2	118.2 (2)	C10—C11—H11A	109.5
N1—C1—C6	123.9 (3)	C10—C11—H11B	109.5
C2—C1—C6	117.9 (2)	H11A—C11—H11B	109.5
C3—C2—C1	121.5 (2)	C10—C11—H11C	109.5
C3—C2—C16	119.5 (3)	H11A—C11—H11C	109.5
C1—C2—C16	119.1 (2)	H11B—C11—H11C	109.5
C2—C3—C4	119.8 (3)	C15—C12—C13	109.7 (2)
C2—C3—H3	120.1	C15—C12—C10	127.5 (2)
C4—C3—H3	120.1	C13—C12—C10	122.8 (2)
C5—C4—C3	120.6 (3)	C14—C13—C12	113.6 (2)
C5—C4—H4	119.7	C14—C13—H13	123.2
C3—C4—H4	119.7	C12—C13—H13	123.2
C4—C5—C6	120.9 (2)	C13—C14—S1	112.9 (2)
C4—C5—H5	119.5	C13—C14—Cl2	127.1 (2)
C6—C5—H5	119.5	S1—C14—Cl2	119.95 (16)
C5—C6—C1	119.2 (3)	C12—C15—Cl1	130.4 (2)
C5—C6—C7	123.9 (2)	C12—C15—S1	113.6 (2)
C1—C6—C7	116.9 (2)	Cl1—C15—S1	115.99 (15)
C8—C7—N2	121.7 (3)	F1—C16—F3	105.6 (2)
C8—C7—C6	118.6 (2)	F1—C16—F2	105.9 (2)
N2—C7—C6	119.7 (2)	F3—C16—F2	105.3 (2)
C7—C8—C9	118.8 (3)	F1—C16—C2	113.8 (2)
C7—C8—H8	120.6	F3—C16—C2	113.7 (2)
C9—C8—H8	120.6	F2—C16—C2	111.8 (2)
N1—C9—C8	126.2 (3)	C9—N1—C1	115.6 (2)
N1—C9—H9	116.9	N3—N2—C7	118.2 (2)
C8—C9—H9	116.9	N3—N2—H1	122.0 (19)
N3—C10—C12	116.4 (2)	C7—N2—H1	119.7 (19)
N3—C10—C11	124.9 (2)	C10—N3—N2	118.0 (2)
C12—C10—C11	118.6 (2)	C14—S1—C15	90.19 (12)
			
N1—C1—C2—C3	177.8 (2)	C10—C12—C13—C14	176.4 (2)
C6—C1—C2—C3	−1.5 (3)	C12—C13—C14—S1	0.8 (3)
N1—C1—C2—C16	−1.6 (3)	C12—C13—C14—Cl2	−177.83 (18)
C6—C1—C2—C16	179.1 (2)	C13—C12—C15—Cl1	−177.8 (2)
C1—C2—C3—C4	−0.2 (4)	C10—C12—C15—Cl1	4.7 (4)
C16—C2—C3—C4	179.2 (2)	C13—C12—C15—S1	1.1 (3)
C2—C3—C4—C5	1.7 (4)	C10—C12—C15—S1	−176.42 (19)
C3—C4—C5—C6	−1.3 (4)	C3—C2—C16—F1	−117.2 (3)
C4—C5—C6—C1	−0.4 (4)	C1—C2—C16—F1	62.2 (3)
C4—C5—C6—C7	−179.8 (2)	C3—C2—C16—F3	121.8 (3)
N1—C1—C6—C5	−177.4 (2)	C1—C2—C16—F3	−58.8 (3)
C2—C1—C6—C5	1.8 (3)	C3—C2—C16—F2	2.7 (3)
N1—C1—C6—C7	1.9 (3)	C1—C2—C16—F2	−177.8 (2)
C2—C1—C6—C7	−178.8 (2)	C8—C9—N1—C1	−1.0 (4)
C5—C6—C7—C8	176.4 (2)	C2—C1—N1—C9	−179.3 (2)
C1—C6—C7—C8	−2.9 (3)	C6—C1—N1—C9	0.0 (3)
C5—C6—C7—N2	−3.4 (4)	C8—C7—N2—N3	1.3 (3)
C1—C6—C7—N2	177.2 (2)	C6—C7—N2—N3	−178.8 (2)
N2—C7—C8—C9	−178.1 (2)	C12—C10—N3—N2	177.7 (2)
C6—C7—C8—C9	2.1 (3)	C11—C10—N3—N2	0.5 (4)
C7—C8—C9—N1	−0.1 (4)	C7—N2—N3—C10	175.9 (2)
N3—C10—C12—C15	9.2 (4)	C13—C14—S1—C15	−0.2 (2)
C11—C10—C12—C15	−173.4 (2)	Cl2—C14—S1—C15	178.60 (17)
N3—C10—C12—C13	−168.0 (2)	C12—C15—S1—C14	−0.6 (2)
C11—C10—C12—C13	9.4 (3)	Cl1—C15—S1—C14	178.47 (16)
C15—C12—C13—C14	−1.2 (3)		
